# Supplementary material for: PfATG18 links V-ATPase assembly to endocytic membrane dynamics in malaria parasites
Source: PLoS Pathog. 2026 Sep 28;22(9):e1014608. doi: 10.1371/journal.ppat.1014608 (PMC13645220; doi:10.1371/journal.ppat.1014608)
Supplement: Supplementary file 1 [file ppat.1014608.s009.pdf]

**Supplemental Table S1.** Diagnostic PCRs performed in this study

| **Mutant** | **PCR**[1] | **Primer** | **Oligo sequence** | **Amplicon size (bp)**[2] |
|---|---|---|---|---|
| *atg18:loxP* | PCR1 | forward | TTTAATAATAAGGTTTTGAAGGGTAAGG | WT 1.711 / Mutant - |
| *atg18:loxP* | PCR1 | reverse | CATTTCGGCTAAGTATAAACCATTC | WT 1.711 / Mutant - |
| *atg18:loxP* | PCR2 | forward | AAAATGTTTATTACCTTGTCATCCG | WT 1.147 / Mutant - |
| *atg18:loxP* | PCR2 | reverse | TTAAAAATAAGCCAAGAAGAAAAACAC | WT 1.147 / Mutant - |
| *atg18:loxP* | PCR3 | forward | TATGTGTTTGTTTTTAAGCTTATTCATG | WT - / Mutant 826 |
| *atg18:loxP* | PCR3 | reverse | ATAACTTCGTATAATGTATGCTATACG | WT - / Mutant 826 |
| *atg18:loxP* | PCR4 | forward | AACTTCGTATAGCATACATTATACG | WT - / Mutant 950 |
| *atg18:loxP* | PCR4 | reverse | GAAGAAAAACACAAAATGAAAAAAAAATAATAAC | WT - / Mutant 950 |
| *atg18:loxP* | PCR5 | forward | AATGAAAAAGGGAAATAAAATAAAAGAATG | Mutant DMSO 1.857 / RAP 801 |
| *atg18:loxP* | PCR5 | reverse | CATCCATCTTGACATTTGGGC | Mutant DMSO 1.857 / RAP 801 |
| *atg18-mNG:loxP* | PCR1 | forward | TTTAATAATAAGGTTTTGAAGGGTAAGG | WT 1.711 / Mutant - |
| *atg18-mNG:loxP* | PCR1 | reverse | CATTTCGGCTAAGTATAAACCATTC | WT 1.711 / Mutant - |
| *atg18-mNG:loxP* | PCR2 | forward | AAAATGTTTATTACCTTGTCATCCG | WT 1.147 / Mutant - |
| *atg18-mNG:loxP* | PCR2 | reverse | TTAAAAATAAGCCAAGAAGAAAAACAC | WT 1.147 / Mutant - |
| *atg18-mNG:loxP* | PCR3 | forward | TATGTGTTTGTTTTTAAGCTTATTCATG | WT - / Mutant 826 |
| *atg18-mNG:loxP* | PCR3 | reverse | ATAACTTCGTATAATGTATGCTATACG | WT - / Mutant 826 |
| *atg18-mNG:loxP* | PCR4 | forward | AACTTCGTATAGCATACATTATACG | WT - / Mutant 950 |
| *atg18-mNG:loxP* | PCR4 | reverse | GAAGAAAAACACAAAATGAAAAAAAAATAATAAC | WT - / Mutant 950 |
| *atg18-mNG:loxP* | PCR5 | forward | AATGAAAAAGGGAAATAAAATAAAAGAATG | Mutant DMSO 2.561 / RAP 801 |
| *atg18-mNG:loxP* | PCR5 | reverse | CATCCATCTTGACATTTGGGC | Mutant DMSO 2.561 / RAP 801 |

[1] PCRs 1-5 indicate the relative primer positions as shown in Figure 1B.
[2] Expected PCR fragments for wild-type (WT) and mutant parasite lines.
