# Supplementary material for: PfATG18 links V-ATPase assembly to endocytic membrane dynamics in malaria parasites
Source: PLoS Pathog. 2026 Sep 28;22(9):e1014608. doi: 10.1371/journal.ppat.1014608 (PMC13645220; doi:10.1371/journal.ppat.1014608)
Supplement: Supplementary file 2 [file ppat.1014608.s010.pdf]

Supplemental Table S2. Oligonucleotides used for guide plasmid generation

Target locus	Orientation[1]	Oligo sequence[2]	Reference
ATG18	forward	attgTACACAAACAACCATAATCC	This study
ATG18	reverse	aaacGGATTATGGTTGTTTGTGTA	This study
FT2	forward	attgAGGAGATATATATATATTGGAA	This study
FT2	reverse	aaacTTCCAATATATATATATCTCCT	This study
Vps26	forward	attgCTTCCTCATCAACAATTATG	This study
Vps26	reverse	aaacCATAATTGTTGATGAGGAAG	This study
Vps29	forward	attgAAGCTCCCGTAACAGAGCCA	This study
Vps29	reverse	aaacTGGCTCTGTTACGGGAGCTT	This study
CRT	forward	attgTGTAATAATTGAATCGACGT	(*5*)
CRT	reverse	aaacACGTCGATTCAATTATTACA	(*5*)
PM2	forward	attgAACGAAAATTTGGAAACCAA	(*5*)
PM2	reverse	aaacTTGGTTTCCAAATTTTCGTT	(*5*)
HRP2	forward	attgTAATACTTTATTTTTTGAGA	(*5*)
HRP2	reverse	aaacTCTCAAAAAATAAAGTATTA	(*5*)
V_1B	forward	attgCCACGTCACCATGCCAACTA	(*5*)
V_1B	reverse	aaacTAGTTGGCATGGTGACGTGG	(*5*)
V_1C	forward	attgTTTAAAGGACACCGAAACGT	(*5*)
V_1C	reverse	aaacACGTTTCGGTGTCCTTTAAA	(*5*)
V_oa	forward	attgATTTCAAAATAAATTTTACA	(*5*)
V_oa	reverse	aaacTGTAAAATTTATTTTGAAAT	(*5*)

[1] Complementary oligonucleotides were annealed, phosphorylated and cloned into the pDC2-Cas9-U6-hDHFRyFCU plasmid using the BbsI restriction site.
[2] Annealing bases are shown in upper case, overhangs in lower case.
