# Supplementary material for: PfATG18 links V-ATPase assembly to endocytic membrane dynamics in malaria parasites
Source: PLoS Pathog. 2026 Sep 28;22(9):e1014608. doi: 10.1371/journal.ppat.1014608 (PMC13645220; doi:10.1371/journal.ppat.1014608)
Supplement: Supplementary file 3 [file ppat.1014608.s011.pdf]

Supplemental Table S3. Repair templates used in this study

atg18:loxP

ATATAAAAATATGAATATATGATGCTTATATAAATAAATAATGAAAAAGGGAAATAAAATAAAAGAATGATTTTTTTAAATTTATATGACATATAATATATACAGAAGAATAAGAG
AAGAAAAAAAAAAAAAAAAGAAAGAAAAAAAAAAAAATTAATAAAGAAAAATAAGCACATAGAAATATATGTGTACTAAATGTAATGTTGATATATTTCATTGTAATAAATATATATTA
TATATATATATTTCCATTTTTATATATTTTACCACATTCATTATAGAAGAAATTAAAAGAAAACAATTTCCGTTTGTTACAGAATAAAAAAAAAAAAAAAATAAATAAAAATAAAA
AAAAAAAAATGGTATCATTAAGATTAGATAATAATAGATATATATCATTTAATCAAGATTACGGATGCTTGTGCATGGCAAATGAAAAGGGATTCAAAATTTATAATACCAACCCA
TTTACCCAAACTTATAGTAGAGGTAAATAAAAAAAATAATATACAATAACTTCGTATAGCATACATTATACGAAGTTATTATATATGTATATATATATATATTTATATATTTTATA
TTCTTTTAGATTTAACAGACAGAAATAAAAATGGACTTTATTTAGCTGAGATGTTATATAGGTGCAATATTTTAGCTATTACTGGTAATAAAAATGACAAAAAGGTAAGTGGGCC
AAGAATGTATTAATTATTTGGGATGACAGACAAATGAGGGAAATTGCTAAATTGACATTCAGTTCAAATATAATTGGTGTAAGGTTGCTTAGAGAGATTATTGTAGTGATACTTGA
ATACAAGTTGTGTATATACAGACTTAAGGACATTATATTGTTGGAAACCTTGAATACAAGTAAGAACGTGTCAGGTTTATGTTGTCTTAGTAACATAGATAAAAATATAATTATAG
CCTACCTTAGTCCAATTAAGGGACGTGTTAACATTCACATTTTTGAGATAAATAGTTCTGAAAATATACACGAGGAGCTTCCTTACATTAATTTCAAAACAAATTTGTCAATTTAT
GCACACGATAATTCTATAGGTTGTATAAATCTTTCAAATGATGGTAAGCTTTTAGTGACTAGTTCTACAAAGGGAACAATTATTAGGCTTTTTAATACCTTCGACGGAACCCTTTT
GAACGAATTCCGTAGGGGAACCAAAAACGCAAAGATATTGTCATTAAATATATCAGAGGATAACAACTGGCTTTGTCTTACTTCTAGTAGAAATACCGTACACGTATTTTCAATTT
ACAAAAAAAAACGTCCTCTTAGGAAGGTTGATATTATTTGCAAAGGTAAAAACGTATCTCCACCTGCCTTACTTAACTACGAGAAAGAGAGTAAAAACAAAAAGTCATCTTTGAAG
TGTCTTCTTCCATGCCATCCTTACCTTAACTCAGAGTGGAGTTTCGCTAGTTACAAATTACCAGGTAAGAAAATTTCATCAATATGTGCATTTGTGAATGATCAGAACTGCATTAT
TGTTATATGTAGTAATGGTATAATTTACAAGTTAAGGTTTAACGAACATATTGGAGGAGATATGTTTAAGATCTCAAGTCACAGTTTTGATGATATCTAAAATATAACTTCGTATA
GCATACATTATACGAAGTTATTTTTTAGTAACCCTCACAGAATAATAAGGACCAAAAATTATATTTCATAAATAAAAAAAATAAATAAATAAATATATATATATATATATATATATT
AAATTAAACTTTTATTTTTTTAATGAACTTTTTAATAATCCACTAAAAGAATTATATATACATATAAATATATATGTATATATATATATATATATATATATATATATATATATATA
TATTTGAACATCTTATTGAGCCCAAATGTCAAGATGGATGAAAAGAAAATAAGAAAAAAAAAAAAGGAAGGTGTCTTATACAAAAATGTACCAAGTTTTGATTAATATATATGTA
ACAAGTTGTTAATGCAGAAAGAAAAAAAAAAAAAAAAAAAAATTAAAAAATTAACACAGAAAAAAAAATATAAAT

atg18-mNG:loxP

ATATAAAAATATGAATATATGATGCTTATATAAATAAATAATGAAAAAGGGAAATAAAATAAAAGAATGATTTTTTTAAATTTATATGACATATAATATATACAGAAGAATAAGAG
AAGAAAAAAAAAAAAAAAAGAAAGAAAAAAAAAAAAATTAATAAAGAAAAATAAGCACATAGAAATATATGTGTACTAAATGTAATGTTGATATATTTCATTGTAATAAATATATATTA
TATATATATATTTCCATTTTTATATATTTTACCACATTCATTATAGAAGAAATTAAAAGAAAACAATTTCCGTTTGTTACAGAATAAAAAAAAAAAAAAAATAAATAAAAATAAAA
AAAAAAAAATGGTATCATTAAGATTAGATAATAATAGATATATATCATTTAATCAAGATTACGGATGCTTGTGCATGGCAAATGAAAAGGGATTCAAAATTTATAATACCAACCCA
TTTACCCAAACTTATAGTAGAGGTAAATAAAAAAAATAATATACAATAACTTCGTATAGCATACATTATACGAAGTTATTATATATGTATATATATATATATTTATATATTTTATA
TTCTTTTAGATTTAACAGACAGAAATAAAAATGGACTTTATTTAGCTGAGATGTTATATAGGTGCAATATTTTAGCTATTACTGGTAATAAAAATGACAAAAAGGTAAGTGGGCC
AAGAATGTATTAATTATTTGGGATGACAGACAAATGAGGGAAATTGCTAAATTGACATTCAGTTCAAATATAATTGGTGTAAGGTTGCTTAGAGAGATTATTGTAGTGATACTTGA
ATACAAGTTGTGTATATACAGACTTAAGGACATTATATTGTTGGAAACCTTGAATACAAGTAAGAACGTGTCAGGTTTATGTTGTCTTAGTAACATAGATAAAAATATAATTATAG
CCTACCTTAGTCCAATTAAGGGACGTGTTAACATTCACATTTTTGAGATAAATAGTTCTGAAAATATACACGAGGAGCTTCCTTACATTAATTTCAAAACAAATTTGTCAATTTAT
GCACACGATAATTCTATAGGTTGTATAAATCTTTCAAATGATGGTAAGCTTTTAGTGACTAGTTCTACAAAGGGAACAATTATTAGGCTTTTTAATACCTTCGACGGAACCCTTTT
GAACGAATTCCGTAGGGGAACCAAAAACGCAAAGATATTGTCATTAAATATATCAGAGGATAACAACTGGCTTTGTCTTACTTCTAGTAGAAATACCGTACACGTATTTTCAATTT
ACAAAAAAAAACGTCCTCTTAGGAAGGTTGATATTATTTGCAAAGGTAAAAACGTATCTCCACCTGCCTTACTTAACTACGAGAAAGAGAGTAAAAACAAAAAGTCATCTTTGAAG
TGTCTTCTTCCATGCCATCCTTACCTTAACTCAGAGTGGAGTTTCGCTAGTTACAAATTACCAGGTAAGAAAATTTCATCAATATGTGCATTTGTGAATGATCAGAACTGCATTAT
TGTTATATGTAGTAATGGTATAATTTACAAGTTAAGGTTTAACGAACATATTGGAGGAGATATGTTTAAGATCTCAAGTCACAGTTTTGATGATATCGGAGCAGGTGCAGGTATGG
TAAGTAAGGGAGAAGAAGACAACATGGCATCATTGCCAGCCACCCACGAGTTGCATATTTTCGGTTCTATTAACGGAGTGGACTTTGATATGGTTGGTCAGGGTACAGGAAATCCT
AATGATGGTTATGAGGAGCTTAATTTGAAATCTACTAAAGGTGACTTGCAGTTTTCACCTTGGATACTTGTTCCACATATAGGTTACGGTTTCACCAGTACCTTCCATATCCAGA
TGGAATGTCTCCATTCCAGGCTGCTATGGTGGATGGAAGTGGTTATCAAGTACATAGGACCATGCAGTTTGAGGACGGTGCATCTTTAACCGTTAACTATAGATATACCTATGAAG
GATCTCACATTAAAGGTGAAGCCCAAGTGAAAGGTACCGGATTTCCTGCTGACGGTCCAGTGATGACTAACAGTTTAACTGCCGCAGACTGGTGCCGTTCTAAAAAGACATATCCA
AACGATAAGACTATAATTTCTACCTTTAAATGGTCATACACAACAGGAAATGGAAAACGTTATAGATCAACTGCAAGGACTACTTATACATTCGCTAAACCAATGGCCGCCAATTA
TCTTAAAAATCAACCTATGTATGTTTTCCGTAAAACTGAATTAAAGCACTCAAAGACAGAACTTAATTTTAAGGAATGGCAAAAAGCATTTACTGACGTAATGGGAATGGATGAGT
TGTATAAGGATATCTAAAATATAACTTCGTATAGCATACATTATACGAAGTTATTTTTTAGTAACCCTCACAGAATAATAAGGACCAAAAATTATATTTCATAAATAAAAAAAATAA
ATAAATAAATATATATATATATATATATATATTAAATTAAACTTTTATTTTTTTAATGAACTTTTTAATAATCCACTAAAAGAATTATATATACATATAAATATATATGTATATAT
ATATATATATATATATATATATATATATATATATATTTGAACATCTTATTGAGCCCAAATGTCAAGATGGATGAAAAGAAAATAAGAAAAAAAAAAAAGGAAGGTGTCTTATACAA
AAATGTACCAAGTTTTGATTAATATATATGTAACAAGTTGTTAATGCAGAAAGAAAAAAAAAAAAAAAAAAAAATTAAAAAATTAACACAGAAAAAAAAATATAAAT

crt-mCh

TTTTTTTTCACAATATACATTTAAATGTTTATGATGGTACAACGTATCATATTTTATAATAATTTTATGCATTCATGTATATTATTTTTACTTTTTAATTTTATAGGGTGATGTTG
TAAGAGAACCAAGATTATTAGATTTCGTAACTTTGGTAAGTGTGAAATTAAAAAATGAATTTTTTTTTTTTTTTTTTATGAACAAAATAATGTGTATATAATATGTGTGTAATATC
AAATGGCTTGTTCGTTCATAAATATTTATATTTTTTTTTTTTTTTTTTTTTTTTTTTTTTTTTACAGTTTGGCTACCTATTTGGTTCTATAATTTACCGTGTAGGAAATATTATCTT
AGAAAGTAATACAAAAATAAGATAAAAAAAATATAATATATAAAATATGTATATTGTTCTTATATATTTGTTCATATATATATATATATATATATATATTTTATATTTCCATCTG
TCTTTTTATTCTATTGTTATAATTTATCATAAATTTTTTTAAATTTGCTTACATTAGGAAAAAAAATGAGAAATGAAGAAAATGAAGATTCCGAAGGAGAATTAACGAATGTGGA
TTCTATTATAACACAAGTTAACGGAGCAGGTGCAGGTATGAAGGTGAGCAAGGGCGAGGAGGATAACATGGCCATCATCAAGGAGTTCATGCGCTTCAAGGTGCACATGGAGGGCT
CCGTGAACGGCCACGAGTTCGAGATCGAGGGCGAGGGCGAGGGCCGCCCCTACGAGGGCACCCAGACCGCCAAGCTGAAGGTGACCAAGGGTGGCCCCCTGCCCTTCGCCTGGGAC
ATCCTGTCCCCTCAGTTCATGTACGGCTCCAAGGCCTACGTGAAGCACCCCGCCGACATCCCCGACTACTTGAAGCTGTCCTTCCCCGAGGGCTTCAAGTGGGAGCGCGTGATGAA
CTTCGAGGACGGCGGCGTGGTGACCGTGACCCAGGACTCCTCCCTGCAGGACGGCGAGTTCATCTACAAGGTGAAGCTGCGCGGCACCAACTTCCCCTCCGACGGCCCCGTAATGC
AGAAGAAGACCATGGGCTGGGAGGCCTCCTCCGAGCGGATGTACCCCGAGGACGGCGCCCTGAAGGGCGAGATCAAGCAGAGGCTGAAGCTGAAGGACGGCGGCCACTACGACGCT
GAGGTCAAGACCACCTACAAGGCCAAGAAGCCCGTGCAGCTGCCCGGCGCCTACAACGTCAACATCAAGTTGGACATCACCTCCCACAACGAGGACTACACCATCGTGGAACAGTA
CGAACGCGCCGAGGGCCGCCACTCCACCGGCGGCATGGACGAGCTGTACAAGGTTAACTAAAATTATATCATTTATTTTTTTATATTTTTTATATTTTTTTTTATTTTTCATGTTTT
TTTCTTTATTTTTTTTTTTTTTTTTTTCCTTTTTTTTTTTAATAAATCATTTTCCTAATATTTATATTTGCTATCGCATTACACTTTATAAGGAATAATGTTGAAGGTATAGAGAT
CTCTTTATTATTATTAAGATGTAAATTTTTTATGAAATTTTGTACTTTTTTAGTATTTATATTATATACACATAAATATATATATATTTATTTATTGATATTTGAAAAAAGAATAA
GGAAATAAAAAAAGGGAAAAAAAAAAAAAGTTAAAAGTATATATATATATATATATATATATATTTATTTATTTATATATATTATTATGTATGTTTATAATTTATATATATATATGTCA
TAAAAAGAAATATAATTATATATATATATATTATATTATATATATATATATAAATTTTTTTAAATTATAAACTATTGTTATTGTTAAATTTAATTTTTAATTCATTAAAAATCAGATT
ACCAAAGATTTTTTCCATTCTTCAATCATGATAATAGAAATGAGAAGAAGCAAAAAAAGAAAAAAAAAAAAAAATTC

crt-mNG

TTTTTTTTCACAATATACATTTAAATGTTTATGATGGTACAACGTATCATATTTTATAATAATTTTATGCATTCATGTATATTATTTTTACTTTTTAATTTTATAGGGTGATGTTG
TAAGAGAACCAAGATTATTAGATTTCGTAACTTTGGTAAGTGTGAAATTAAAAAATGAATTTTTTTTTTTTTTTTTTATGAACAAAATAATGTGTATATAATATGTGTGTAATATC
AAATGGCTTGTTCGTTCATAAATATTTATATTTTTTTTTTTTTTTTTTTTTTTTTTTTTTTTTACAGTTTGGCTACCTATTTGGTTCTATAATTTACCGTGTAGGAAATATTATCTT
AGAAAGTAATACAAAAATAAGATAAAAAAAATATAATATATAAAATATGTATATTGTTCTTATATATTTGTTCATATATATATATATATATATATATATTTTATATTTCCATCTG
TCTTTTTATTCTATTGTTATAATTTATCATAAATTTTTTTAAATTTGCTTACATTAGGAAAAAAAATGAGAAATGAAGAAAATGAAGATTCCGAAGGAGAATTAACGAATGTGGA
TTCTATTATAACACAAGTTATCGGAGCAGGTGCAGGTATGGTAAGTAAGGGAGAAGAAGACAACATGGCATCATTGCCAGCCACCCACGAGTTGCATATTTTCGGTTCTATTAACG
GAGTGGACTTTGATATGGTTGGTCAGGGTACAGGAAATCCTAATGATGGTTATGAGGAGCTTAATTTGAAATCTACTAAAGGTGACTTGCAGTTTTCACCTTGGATACTTGTTCCA
CATATAGGTTACGGTTTCACCAGTACCTTCCATATCCAGATGGAATGTCTCCATTCCAGGCTGCTATGGTGGATGGAAGTGGTTATCAAGTACATAGGACCATGCAGTTTGAGGA
CGGTGCATCTTTAACCGTTAACTATAGATATACCTATGAAGGATCTCACATTAAAGGTGAAGCCCAAGTGAAAGGTACCGGATTTCCTGCTGACGGTCCAGTGATGACTAACAGTT
TAACTGCCGCAGACTGGTGCCGTTCTAAAAAGACATATCCAAACGATAAGACTATAATTTCTACCTTTAAATGGTCATACACAACAGGAAATGGAAAACGTTATAGATCAACTGCA
AGGACTACTTATACATTCGCTAAACCAATGGCCGCCAATTATCTTAAAAATCAACCTATGTATGTTTTCCGTAAAACTGAATTAAAGCACTCAAAGACAGAACTTAATTTTAAGGA
ATGGCAAAAAGCATTTACTGACGTAATGGGAATGGATGAGTTGTATAAGGATAACTAAAATTATATCATTTATTTTTTTATATTTTTTATATTTTTTTTTATTTTTCATGTTTTT
CTTTATTTTTTTTTTTTTTTTTTTCCTTTTTTTTTTTAATAAATCATTTTCCTAATATTTATATTTGCTATCGCATTACACTTTATAAGGAATAATGTTGAAGGTATAGAGATCTC
TTTATTATTATTAAGATGTAAATTTTTTATGAAATTTTGTACTTTTTTAGTATTTATATTATATACACATAAATATATATATATTTATTTATTGATATTTGAAAAAAGAATAAGGA
AATAAAAAAAGGGAAAAAAAAAAAAAGTTAAAAGTATATATATATATATATATATATATATTTATTTATTTATATATATTATTATGTATGTTTATAATTTATATATATATATGTCATAA
AAAGAAATATAATTATATATATATATATTATATTATATATATATATATAAATTTTTTTAAATTATAAACTATTGTTATTGTTAAATTTAATTTTTAATTCATTAAAAATCAGATTACC
AAAGATTTTTTCCATTCTTCAATCATGATAATAGAAATGAGAAGAAGCAAAAAAAGAAAAAAAAAAAAAAATTC

pm2-mCh

TATCAATAGGTTCAGTAGATCCAATTGTTGTTGAATTAAAAAACCAAAACAAAATTGAAAATGCTCTTTTCACCTTTTACTTACCTGTACATGATAAACATACAGGATTCTTAACC
ATTGGTGGTATTGAAGAAAGATTTTATGAAGGACCACTAACTTACGAAAAATTAAACCACGATTTATATTGGCAAAAACTTTAGATGCACACGTTGGAAATATAATGTTAGAAAA
AGCAAACTGTATTGTAGATAGTGGTACTAGTGCCATTACTGTACCAACTGACTTTTTAAATAAAATGTTGCAGAATTTAGATGTTATCAAAGTCCCATTCTTACCTTTCTATGTAA
CTCTTTGTAACAACAGCAAATTACCAACTTTTGAATTTACCTCAGAAAATGGTAAATACACATTAGAACCTGAATACTACCTTCAACACATAGAAGATGTTGGTCCAGGATTATGT
ATGCTTAATATCATAGGATTAGATTTTCCAGTACCAACCTTTATTCTAGGTGACCCATTCATGAGAAAATATTTTACCGTCTTTGATTATGATAATCAGAGTGTTGGTATTGCTCT
TGCTAAAAAGAATTTAGTTATCGGAGCAGGTGCAGGTATGAAGGTGAGCAAGGGCGAGGAGGATAACATGGCCATCATCAAGGAGTTCATGCGCTTCAAGGTGCACATGGAGGGCT

CCGTGAACGGCCACGAGTTCGAGATCGAGGGCGAGGGCGAGGGCCGCCCCTACGAGGGCACCCAGACCGCCAAGCTGAAGGTGACCAAGGGTGGCCCCCTGCCCTTCGCCTGGGAC
ATCCTGTCCCCTCAGTTCATGTACGGCTCCAAGGCCTACGTGAAGCACCCCGCCGACATCCCCGACTACTTGAAGCTGTCCTTCCCCGAGGGCTTCAAGTGGGAGCGCGTGATGAA
CTTCGAGGACGGCGGCGTGGTGACCGTGACCCAGGACTCCTCCCTGCAGGACGGCGAGTTCATCTACAAGGTGAAGCTGCGCGGCACCAACTTCCCCTCCGACGGCCCCGTAATGC
AGAAGAAGACCATGGGCTGGGAGGCCTCCTCCGAGCGGATGTACCCCGAGGACGGCGCCCTGAAGGGCGAGATCAAGCAGAGGCTGAAGCTGAAGGACGGCGGCCACTACGACGCT
GAGGTCAAGACCACCTACAAGGCCAAGAAGCCCGTGCAGCTGCCCGGCGCCTACAACGTCAACATCAAGTTGGACATCACCTCCCACAACGAGGACTACACCATCGTGGAACAGTA
CGAACGCGCCGAGGGCCGCCACTCCACCGGCGGCATGGACGAGCTGTACAAGGATAACTAAAAACGATTATTAGGATACTAAAGAAAAAACATAATTATATAAGTATAATTATATA
TATTCACATTTTTTATATATGAATATAAGAAAACATATTTTATCCATAATGAACCATTTCATAGAAATTTAAATAATTAAAAAATATTTTTTATTTTTTTTTATTTTTATTTTTAT
TTTTTTATTTTATTTTTTTTTTTTTTTTATTTTTTTTTTTTACATTCTCCGTATTCTTGTAAAAGAATATAATGATAATAAAAACATTTTTTTATTATGATTTTATAATTTTTTATA
TTTAATTTTTTTTATTTTTTTCATAGCATTAATTAATATATATATATATATATATATTAGTTATATTTGTGAAATAACTTTTCTTTAATAACCTAACACAAGAGTGAGTTCATAAAAT
TTTATATTTTGCCATATATATATATATATATATATATATATATATATATATATATATATATATATACATATATATATTTATGTATATATGTGTTTTTCCAATATTGGTTCACATTTCT
TCTTTTTTCACTCGAAAATATTTGACTTATTATATTTTATATTATATATATAAAAAAAAAAAAAAAAAATTGTTAAAG

ft2-mCh

TTGCTTATTTCTCTGGTTATTTTTTAGAAAAGATGCCTAGAGAATATATATTTATAGCCACTTCCATATTTCCGTTAATTATATCTTTATCATGTTTATTTTTAAAAGAAAAAGAA
TATTCCACAAACCGAAATATATTTAACCAGTTATCAGATTTAATTAAATTTATAAATACACCTATATTTTTAGGACCATTCTTATATATATTTGTTTATATGTCTGGTCCAGATTA
TGATGATGCTTTTTTTTTTTTCTGTACAAATAAATTAGGATTCAGACCATCGTTTATGGGGACATTAAGATTAACATATGGTATAGCCTCTTTAATAGGAATAATTATTTATCGTG
TTTTCCTAAAAAATTGTAGTTTAAGAAAAACATTAATAATAACAACTTTAGTTAGTTTCCCTATTTACATTAGCCCAATTATACTTACTGAACACATAAATAAGTTTTTGGGAATT
TCAAACGAGTTGTTTGTTCTTTCAGGAGGATTTCTTATTGAAGCTATAACCGAAATTCAACTTTTACCATTGTTCATTTTAACAGCTAATATTTGTCAGCCTGGTCTTGAAGCATC
AGTTTTTGCTACTATTTTGTCAGTGAAAAACCTTGGTTCTTTGACCAAAAAAGGAACCTCTTCATTTATTACCTACTTGATGAAAATTGATTCTTATAATTTCGACAACTTGTCAT
TGTATATATTAACCTGTGGTTTTTTCCTTCTTTTCTCATTGACTTTGGTGCCACTTTTGCCTAATGAGGAACACATTGAGAAGTTGAAAAACAAGGAGACCTCAAAGGGAGGATCC
GGAGCAGGTGCAGGTATGAAGGTGAGCAAGGGCGAGGAGGATAACATGGCCATCATCAAGGAGTTCATGCGCTTCAAGGTGCACATGGAGGGCTCCGTGAACGGCCACGAGTTCGA
GATCGAGGGCGAGGGCGAGGGCCGCCCCTACGAGGGCACCCAGACCGCCAAGCTGAAGGTGACCAAGGGTGGCCCCCTGCCCTTCGCCTGGGACATCCTGTCCCCTCAGTTCATGT
ACGGCTCCAAGGCCTACGTGAAGCACCCCGCCGACATCCCCGACTACTTGAAGCTGTCCTTCCCCGAGGGCTTCAAGTGGGAGCGCGTGATGAACTTCGAGGACGGCGGCGTGGTG
ACCGTGACCCAGGACTCCTCCCTGCAGGACGGCGAGTTCATCTACAAGGTGAAGCTGCGCGGCACCAACTTCCCCTCCGACGGCCCCGTAATGCAGAAGAAGACCATGGGCTGGGA
GGCCTCCTCCGAGCGGATGTACCCCGAGGACGGCGCCCTGAAGGGCGAGATCAAGCAGAGGCTGAAGCTGAAGGACGGCGGCCACTACGACGCTGAGGTCAAGACCACCTACAAGG
CCAAGAAGCCCGTGCAGCTGCCCGGCGCCTACAACGTCAACATCAAGTTGGACATCACCTCCCACAACGAGGACTACACCATCGTGGAACAGTACGAACGCGCCGAGGGCCGCCAC
TCCACCGGCGGCATGGACGAGCTGTACAAGGAATTCTAAATAATGTTCAAAAAAATAAAATAAAATAAAATAAAATACATATAAACACACATATAAACACACATATAAACACACAT
ATAAACACACATATAAACACACATATAAACACACATATAAACACACATATAAACACACATATAAACACACATATAAACACACATATAAACACACATATAAACACACATACATACAT
ACATACATACACACATACATATGTATATATAAATATATAGATATATATTTATATTTATATTTATTTAATATTATATGTATAAATTTCATATTTATTATTATTTTTTTTTTTTTTTT
ATCATCACATTTGTCAAAAAAAAAAAAAAAAAGGGGGTCCATCAAAAAAAAAAAAAAAATTTGTTTTGTTTTCATAAAATATATGAAAGTA

vps26-mCh

AATATACAAAAAGAAATTGATTTCATTGTTCAGAATTTATGTATACCACCTGAAATTAATAATACCATTAAAATGGAAGTAGGCATAGAAGATTGTCTACATATCGAATTTGAATA
TGATAAATCCAAATATCACTTAAAAGATGTAGTTGTTGGAAAAGTTTATTTTCTTCTAGTAAGAATTAAAATTAAACACATGGAATTAGATATCATAAAAATGGAAACCTCAGGTG
TTGGCAAAAATTATACTACAGAAACTGTAACCTTGTCAAAATTTGAAATAATGGACGGATCTCCAATCAAATCGGAGTGTATTCCTGTGAGATTATATCTAAGTGGTTTCGACCTA
ACGCCCACGTATAAGAATATTCAAAATAAATTCTCGGTCAAATATTATATTAATCTCATTATAGTAGACGAAGAGGAGAAGATATTTTAAGAAACAAGAAATATTTTTGTGGAG
AAAAAAGATGGGAGAGCTCGGAGCAGGTGCAGGTATGAAGGTGAGCAAGGGCGAGGAGGATAACATGGCCATCATCAAGGAGTTCATGCGCTTCAAGGTGCACATGGAGGGCTCCG
TGAACGGCCACGAGTTCGAGATCGAGGGCGAGGGCGAGGGCCGCCCCTACGAGGGCACCCAGACCGCCAAGCTGAAGGTGACCAAGGGTGGCCCCCTGCCCTTCGCCTGGGACATC
CTGTCCCCTCAGTTCATGTACGGCTCCAAGGCCTACGTGAAGCACCCCGCCGACATCCCCGACTACTTGAAGCTGTCCTTCCCCGAGGGCTTCAAGTGGGAGCGCGTGATGAACTT
CGAGGACGGCGGCGTGGTGACCGTGACCCAGGACTCCTCCCTGCAGGACGGCGAGTTCATCTACAAGGTGAAGCTGCGCGGCACCAACTTCCCCTCCGACGGCCCCGTAATGCAGA
AGAAGACCATGGGCTGGGAGGCCTCCTCCGAGCGGATGTACCCCGAGGACGGCGCCCTGAAGGGCGAGATCAAGCAGAGGCTGAAGCTGAAGGACGGCGGCCACTACGACGCTGAG
GTCAAGACCACCTACAAGGCCAAGAAGCCCGTGCAGCTGCCCGGCGCCTACAACGTCAACATCAAGTTGGACATCACCTCCCACAACGAGGACTACACCATCGTGGAACAGTACGA
ACGCGCCGAGGGCCGCCACTCCACCGGCGGCATGGACGAGCTGTACAAGCCGCGGTAGAAAAAAGAAAATGACAATAATATAAATATAAATATATAAATATATAAATATATATATAT
ATATATATATATATATATTTATTTATTTATTTGTTGAAGGGGTCTTGTAATTTTTTTTATATAAACAATGTGAAGCTTTTTCTTTTTTTTTTTATGAACTGTTCATATTCTT
TTTTTTTTTTTTATATAAAATGGCTTTTTTTTTTTTTTTTTTTTCTTATTTACATGATCAAGTCATATGGTTCTAACATATTTCTGAACATGTTCATAATATATAAATTTATTT
TTTTTTATATTTTTTTTTGTTTTTCTATATTTTTAATTTTTTATGATTAATATAATTTAGCATAATTTGCATTCTCTAATAATACTGCTACAAATAATATATATATA

vps29-mCh

GATATTGGGGAACTAGTTTTATTGATAGGAGATTTTCATTCTCCTATTCGTAATTTAGGACTTCCTGATTGTTTTAAGGAACTTTTAAAGACAGATAAAATAAAACATGTTTTATG
TACAGGAAATGTAGGATGTAATGAGAATTTGGAATTACTTAAAACATTGCTGACTCAGTACATATAACAAAAGGTGATATGGATGATAATTTTGATTTCCCAGAAGATATTACAT
TATGTATAGGAGATTTTAAAATATCTTTAATTCATGGACATCAAATAATTCCATGGGGAGATATGAATGCTCTTTTACAATGGCAAAAAAAATATGATAGTGATATTATTATAAGT
GGACATACACATAAAAATTCGATTGTTCAATATGAGGGCAAATATTTTATTAATCCTGGTTCTGTAACTGGAGCCTTTCAACCTTGGTTGAGTGAGCCTACTCCAACATTCATACT
TATGGCTGTGGCAAAAAGTAATATAGTGTTATATGTTTACGAGGAAAAGAACGGAAAGACTAACGTGGAGATGTCTGAGTTACACAAGAGTACAGTTATAGAGCTCGGAGCAGGTG
CAGGTATGAAGGTGAGCAAGGGCGAGGAGGATAACATGGCCATCATCAAGGAGTTCATGCGCTTCAAGGTGCACATGGAGGGCTCCGTGAACGGCCACGAGTTCGAGATCGAGGGC
GAGGGCGAGGGCCGCCCCTACGAGGGCACCCAGACCGCCAAGCTGAAGGTGACCAAGGGTGGCCCCCTGCCCTTCGCCTGGGACATCCTGTCCCCTCAGTTCATGTACGGCTCCAA
GGCCTACGTGAAGCACCCCGCCGACATCCCCGACTACTTGAAGCTGTCCTTCCCCGAGGGCTTCAAGTGGGAGCGCGTGATGAACTTCGAGGACGGCGGCGTGGTGACCGTGACCC
AGGACTCCTCCCTGCAGGACGGCGAGTTCATCTACAAGGTGAAGCTGCGCGGCACCAACTTCCCCTCCGACGGCCCCGTAATGCAGAAGAAGACCATGGGCTGGGAGGCCTCCTCC
GAGCGGATGTACCCCGAGGACGGCGCCCTGAAGGGCGAGATCAAGCAGAGGCTGAAGCTGAAGGACGGCGGCCACTACGACGCTGAGGTCAAGACCACCTACAAGGCCAAGAAGCC
CGTGCAGCTGCCCGGCGCCTACAACGTCAACATCAAGTTGGACATCACCTCCCACAACGAGGACTACACCATCGTGGAACAGTACGAACGCGCCGAGGGCCGCCACTCCACCGGCG
GCATGGACGAGCTGTACAAGCCGCGGTGATATATATATATATATAAGTACATTTATATTTATTTTTTCTTATTTGTTTATATATATATATATATATATTTTACTTGAAACTAATTTTAA
TTAAAATTTTTATATTTAAACCTTCCTTTTATTTTTTTAAAATATTATATTCCTTTTCCAACATCTCTTTTACACGTCATTTATGTAAATTCAAAAAAATGAAAATAAAATAATTC
AACTAATAAAAGGAAATATATATAATTATTTATTATGCATAATTTGAAAGGGTTTTTTTCTCACATATTTAAATTATAAGAGAATATATGTTGCTAATTTATATTATAGAAAATC
GACATAGGAATTATAAAACCTCATTAATACATATATAAAACGTGTAATCAAATTATTTGTCATCAAAAATATAAAACCGTGGT

hrp2-mCh

TTATTTAAGCATTTATAATTTTTCATCTACTTTGCTTTTAAGTTTCTCCATTTTTAATACTTCAATTTTCTTTTCTTTTTTTTTAAATAAATCAATATAATTATAATTAATTTTCC
CTTTTTATATAATTAAAAGTATAGAACAATTAAATAATTTTATATAATAATAATATATATATATATATATATATATATGTACATTATTTATGAAAAAAAGTATATAATGAATTATATA
TAATGTTTATTTTTCACTTCATGTATTTATGTATGCAGAACTTTTTTTTTGTATATGCAAAAAATTAGTATTCTATATTTTATATATATCATATATTATACAATTATATTTATACA
TTTTGTTATTATTTCTTTTTCTATTTATATAGTTATTTAATAAAAATGGTTAGTTTCTCTAAAAACAAGGTGTTGTCTGCTGCAGTTTTCGCTTCAGTTTTATTGTTGGACAATA
ATAACTCTGCATTTAATAACAATCTTTGCTCAAAAAACGCCAAAGGACTTAATTTAAACAAACGTCTTTTACACGAAACACAAGCCCACGTGGATGACGCACATCACGCCCATCAT
GTTGCCGATGCCCATCACGCTCATCATGTTGCTGATGCTCATCATGCACATCATGTGGCTGATGCACACCACGCACACCATGCAGCTGACGCACATCATGCCCATCACGCCGCAGA
CGCTCACCACGCCCACCATGCTGCTGACGCCCACCACGCCCATCACGCACATCATGCTGCCGATGCTCATCATGCTCATCATGCAGCCGACGCTCATCATGCTCATCACGCTGCTG
ATGCTCACCATGCTCACCACGCTGCTGATGCCCACCATGCTCATCATGCAGCAGACGCTCATCACGCCCATCACGCAAGTGACGCACATCACGCACACCACGCCGCATATGCACAC
CACGCCCATCACGCAAGTGATGCTCACCACGCTGCAGATGCCCATCATGCTGCCTATGCTCATCATGCCCATCATGCTGCTGACGCACACCATGCCGCAGATGCACACCATGCCAC
TGATGCTCACCATGCCGCCGATGCTCATCACGCTGCTGATGCACACCACGCAGCCGACGCTCACCACGCAACTGATGCCCACCACGCACACCATGCAGCTGATGCACACCACGCCA
CCGACGCCCATCATGCACACCATGCCGCCGACGCTCACCACGCTGCTGCCCATCATGCAACAGATGCACATCATGCTGCAGCTCACCATGCAACTGACGCTCACCATGCCGCTGCT
CACCACGAGGCCGCCACACACTGTCTTAGGCATGGATCCGGAGCAGGTGCAGGTATGAAGGTGAGCAAGGGCGAGGAGGATAACATGGCCATCATCAAGGAGTTCATGCGCTTCAA
GGTGCACATGGAGGGCTCCGTGAACGGCCACGAGTTCGAGATCGAGGGCGAGGGCGAGGGCCGCCCCTACGAGGGCACCCAGACCGCCAAGCTGAAGGTGACCAAGGGTGGCCCCC
TGCCCTTCGCCTGGGACATCCTGTCCCCTCAGTTCATGTACGGCTCCAAGGCCTACGTGAAGCACCCCGCCGACATCCCCGACTACTTGAAGCTGTCCTTCCCCGAGGGCTTCAAG
TGGGAGCGCGTGATGAACTTCGAGGACGGCGGCGTGGTGACCGTGACCCAGGACTCCTCCCTGCAGGACGGCGAGTTCATCTACAAGGTGAAGCTGCGCGGCACCAACTTCCCCTC
CGACGGCCCCGTAATGCAGAAGAAGACCATGGGCTGGGAGGCCTCCTCCGAGCGGATGTACCCCGAGGACGGCGCCCTGAAGGGCGAGATCAAGCAGAGGCTGAAGCTGAAGGACG
GCGGCCACTACGACGCTGAGGTCAAGACCACCTACAAGGCCAAGAAGCCCGTGCAGCTGCCCGGCGCCTACAACGTCAACATCAAGTTGGACATCACCTCCCACAACGAGGACTAC
ACCATCGTGGAACAGTACGAACGCGCCGAGGGCCGCCACTCCACCGGCGGCATGGACGAGCTGTACAAGGAATTCTAAATTTATTTAATAATAGATTAAAAATATTATAAAAATAA
AAACATAAACACAGAAATTACAAAAAAAATACATATGAATTTTTTTTTTGTAATCTTCCTTATAAATATAGAATAATGAATCATATAAAACATATCATTATTCATTTATTTACATT
TAAAATTATTGTTTCAGTATCTTTAATTTATTATGTATATATAAAAATAACTTACAATTTTATTAATAAACAATATATGTTTATTAATTCATGTTTTGTAATTTATGGGATAGCGA
TTTTTTTTACTGTCTGTATTTTTCTTTTTTAATTATGTTTTAATTGTATTTTATTTTTATTATTGTTCTTTTTATAGTATTATTTTAAAACAAAATGTATTTTCTAAGAACTTATA
ATAATAATAAATAT

v_1B-mNG

CGGCAAGAGAAGAAGTACCAGGTAGAAGAGGATATCCAGGATATATGTATAGTGATTTATCCACCATATATGAAAGAGCTGGAAGAGTTGAAGGACGTAATGGTAGTATAACACAA
TTTCCTATATTAACAATGCCTAATGATGATATTACACATCCAATACCTGATTTAACCGGATATATTACAGAAGGTCAAATATTTGTAGACAGAAATTTATATAATAGACAAATATA
TCCTCCAATCAATGTCTTACCATCTTTATCGCGTTTAATGAAAAGTGGTATTGGACATAATATGACCAGAATCGATCATCCATATGTATCTGATCAATTATATAGTAATTATGCTA
TTGCTCAAGATGTAAAAGCAATGAAAGCAGTAATTGGTGAAGAAGCACTTTCAAATGATGATATTCTATATCTTGAATTCTTAGATAAATTCGAAAAGAGATTTATTACACAAAAT

ACATATGAATGTAGAGATATTTATCAATCATTGGATATCGCATGGGAACTTTTAAGAATCTTTCCAGAAGATATGCTTAAAAAAATTAAAACAGACATTTTATCCAAATATTATCC
TCGACATCACGCAAACGTTATCGGAGCAGGTGCAGGTATGGTAAGTAAGGGAGAAGAAGACAACATGGCATCATTGCCAGCCACCCACGAGTTGCATATTTTCGGTTCTATTAACG
GAGTGGACTTTGATATGGTTGGTCAGGGTACAGGAAATCCTAATGATGGTTATGAGGAGCTTAATTTGAAATCTACTAAAGGTGACTTGCAGTTTTCACCTTGGATACTTGTTCCA
CATATAGGTTACGGTTTTCACCAGTACCTTCCATATCCAGATGGAATGTCTCCATTCCAGGCTGCTATGGTGGATGGAAGTGGTTATCAAGTACATAGGACCATGCAGTTTGAGGA
CGGTGCATCTTTAACCGTTAACTATAGATATACCTATGAAGGATCTCACATTAAAGGTGAAGCCCAAGTGAAAGGTACCGGATTTCCTGCTGACGGTCCAGTGATGACTAACAGTT
TAACTGCCGCAGACTGGTGCCGTTCTAAAAAGACATATCCAAACGATAAGACTATAATTTCTACCTTTAAATGGTCATACACAACAGGAAATGGAAAACGTTATAGATCAACTGCA
AGGACTACTTATACATTCGCTAAACCAATGGCCGCCAATTATCTTAAAAATCAACCTATGTATGTTTTCCGTAAAACTGAATTAAAGCACTCAAAGACAGAACTTAATTTTAAGGA
ATGGCAAAAAGCATTTACTGACGTAATGGGAATGGATGAGTTGTATAAGGATAACTAAGGAAAATCAAAAAAACATATATGAAATGATAAATTGTGCCTAAAAAAAAAAAAAAAAAA
AATACATATATATATATATATATATATATATAATATATATGTTTATACATATATGTAATAATTTATTTTTTTTCAAGGTGTTAAAAAAATGTACCCCCACTTAAATTTTTTTTTTT
TTGAATAATCTTATCTATTTAATTCATTTTTAAATAAAAAAAAAAAAAAGAAACAACCTTTTCATTATATATGTTTGTATATATCTATATGTATTGTTTTATTTTATTCATTTAAT
AATAATAGTATATCCGTTACATCATTTTTTTTTTTTTTTTTTTTTTTTTTATATATATTCCAAAATTTTATTGTTCTTATATATATAATATATATATATATATATATATATTAAACAA
ATAAGTAGCTTATTATATATCATATTATATATAATATTATGTAGAATTTTTTTTTTTTTTTTTCTTAATTTTGTTTAATAATTTAAAATTTTCTAATAACTAAAAAATAAGTCAAAT
AAAATCAAAAAAAAATAAAATAATAAATGCATAAAAAAATTAAACAATAAATGTACATATATATTTGTATATTT

---

v_1C-mNG

TATAGAAACAGAATATCTAACAACACTTATAGCTTATGTACCTAAAAATTCTATAGATGATTGGTTAAATAATTATGAAAAATTTTCATCCTATGTTGTACCTAGATCTACAGAAC
AATTTAAAGATTTAATAGATAAAGATGGAAATACATTATGGAAAGTTTTTGTTTTTAAGAAATTTGCAGAAGATTTTAAAAAAGAAGCAAAAGTTAAAAAATTTGTTGTAAAATCA
TTTAAATATGATGAAAAACAATATAATGATATGATGGAATCGAGAACAAAAGTAGAAGCAGAAATCATAAGACAAGAAACTTTTCTAAGACGCATGTGCTTAGCCGCTTTTTCAGA
TATATTTATTGCATTCATTCATATTAATATACTTCGAGTCTTTTGTGAATCTGTATTACGATTTGGTGTTCCACCTAATTTTGCTTCATTTAGTATAAGAATTAATGGAGAAAGTA
AAGAAAAAAAAGTCAGAAAAAAATTATACGACATTTTTTCATCATCTGATTCTATAGGAAAGAATTATATAAAAAGATCTGATGAAAATGATGAAGAAATATATCCATATGTATCA
GTATCATTTAAGATAGATATCGGAGCAGGTGCAGGTATGGTAAGTAAGGGAGAAGAAGACAACATGGCATCATTGCCAGCCACCCACGAGTTGCATATTTTCGGTTCTATTAACGG
AGTGGACTTTGATATGGTTGGTCAGGGTACAGGAAATCCTAATGATGGTTATGAGGAGCTTAATTTGAAATCTACTAAAGGTGACTTGCAGTTTTCACCTTGGATACTTGTTCCAC
ATATAGGTTACGGTTTTCACCAGTACCTTCCATATCCAGATGGAATGTCTCCATTCCAGGCTGCTATGGTGGATGGAAGTGGTTATCAAGTACATAGGACCATGCAGTTTGAGGAC
GGTGCATCTTTAACCGTTAACTATAGATATACCTATGAAGGATCTCACATTAAAGGTGAAGCCCAAGTGAAAGGTACCGGATTTCCTGCTGACGGTCCAGTGATGACTAACAGTTT
AACTGCCGCAGACTGGTGCCGTTCTAAAAAGACATATCCAAACGATAAGACTATAATTTCTACCTTTAAATGGTCATACACAACAGGAAATGGAAAACGTTATAGATCAACTGCAA
GGACTACTTATACATTCGCTAAACCAATGGCCGCCAATTATCTTAAAAATCAACCTATGTATGTTTTCCGTAAAACTGAATTAAAGCACTCAAAGACAGAACTTAATTTTAAGGAA
TGGCAAAAAGCATTTACTGACGTAATGGGAATGGATGAGTTGTATAAGGATATCTGAAATTGGAAAGGCTATAAATTGGGTATACCAATATGTATAAATGTATATAAATGTATATA
AATGTATATATGCATATATATATATATATATATATATATATATGTGTATGTTATTTTTTATGTGTTAATGAACCAATTTATTATTATTACAACCAAATCCAGGAATATCAACCC
ATGATATGTGCTACACATGTTTAAAAGTATTAGATACCAATATGAAGGTGAAGAAGAAAAGAATTATTTTTGTAAGAATAATATTTTTTTACGCAACTATTATATTTTACATGTAA
CTATTACTTTATTTTATTTTTTCCATTTATTAAAAAAAAAAGTAGCACAAATTAGAAGAAGATATTATTTTATTTGTACCCATGATTGTTACAGTACTCTACTTTCTAATATATAA
GCTAAAAATATAGTAAAAATAATTAATTGTTAACATTAATTTTATATGAGAACTTATAATAGAAAAATATAATTATATATATATATATATATATATATATATTTATTAATTTTATA
TATATTTTAATTGTTATCTTTTTTCTTTTTTTTTTTTTTTTTTTTTTTTTTCCGCTTTATTAATAAGATGAACT

---

v_oa-mNG

ACAACCTGACGATGTATATGATCAATATGACGAATACGATGATGATGATGATGATGATAATTATGACGAAAATGAATATTTAAATAAAAAAAAAAAAAAAAGAACAGATGATGATA
TAGAAGCTCATTTATTAAGTTCTACATATGAAGAGAAAGGATTACAAAGTTCATCAGCAGCCATGAGAGGAGGAGGAGCAGGAGAAGAGAATCATCATGAAGAAAACATTTCAGAA
ATATGGATTGAACAATTAATAGAAACAATTGAATTTATATTAGGATTAATTAGTAACACAGCATCATATTTAAGATTATGGGCATTATCATTAGCTCATCAACAATTATCATTTGT
CTTTTTTGAGCAAACCATTTTAAATTCCTTAAAGAGAAATAGTTTTATGTCTGTATTAATTAACTTAATATTATTTTCTCAATTATTTTCAATACTTACCATAGCTGTTATATTAT
GTATGGACACACTGGAATGTTTCTTACATTCATTAAGATTGCAGTGGGTTGAATTCCAGAACAAGTTTTATAAAGGAGATGGTATTCCCTTCAAGCCTTTCAACATAAAGAAGTTG
TTGAACGAAAATGAGGATATCGGAGCAGGTGCAGGTATGGTAAGTAAGGGAGAAGAAGACAACATGGCATCATTGCCAGCCACCCACGAGTTGCATATTTTCGGTTCTATTAACGG
AGTGGACTTTGATATGGTTGGTCAGGGTACAGGAAATCCTAATGATGGTTATGAGGAGCTTAATTTGAAATCTACTAAAGGTGACTTGCAGTTTTCACCTTGGATACTTGTTCCAC
ATATAGGTTACGGTTTTCACCAGTACCTTCCATATCCAGATGGAATGTCTCCATTCCAGGCTGCTATGGTGGATGGAAGTGGTTATCAAGTACATAGGACCATGCAGTTTGAGGAC
GGTGCATCTTTAACCGTTAACTATAGATATACCTATGAAGGATCTCACATTAAAGGTGAAGCCCAAGTGAAAGGTACCGGATTTCCTGCTGACGGTCCAGTGATGACTAACAGTTT
AACTGCCGCAGACTGGTGCCGTTCTAAAAAGACATATCCAAACGATAAGACTATAATTTCTACCTTTAAATGGTCATACACAACAGGAAATGGAAAACGTTATAGATCAACTGCAA
GGACTACTTATACATTCGCTAAACCAATGGCCGCCAATTATCTTAAAAATCAACCTATGTATGTTTTCCGTAAAACTGAATTAAAGCACTCAAAGACAGAACTTAATTTTAAGGAA
TGGCAAAAAGCATTTACTGACGTAATGGGAATGGATGAGTTGTATAAGGATATCTAACAAGAACAAAATCATAAAAGTGTACCATCAACTGTGTAAGTCGTTATAAGCACACACAT
AAATAAAAATAAAAATGAACATATAAATGAATCAATATATATAAATATATATATATATATATATATATATATATATATATATGTATGTATGTATGTTTGTTTGTTTTTATTTATGT
GTGCTCATTTTTTTTTTTTTTTTCAATTTATGTAATTTAGATATTTTCAAGTTAAAATATTTATGTTGTATTCCTTACGTTTTATGTTTATTTATATAATTAATTCTTTTATTATA
TATTATATTATATTTTATTCCTTTTACTTTTACTTTTTCTTTTTTTTTTTTTTTTTTTTTTTTTTTTTTGTTAACATTATAAAAAAATTAAAAACAGCTAGCCTTTTTTTGAAGGGG
TAAAAACCTGCATGCAAAAAAAAAAAAAAAAAAAAAAAAATATGAACATATAAAGTGCATTCTGTTATATATATATATATATATATATATATATAATTGCATAATCTAGTTGTGTATTTT
TCTCATTTGTTAACATTACAATGTTGACCACCTTATATAAAAAACATATAAGCTTTTTATAAAGAAAAGATAA

Color code: Homology regions, recodonized sequence, intron, *loxP*, linker, mNG, mCh, stop codon
