# Supplementary material for: PfATG18 links V-ATPase assembly to endocytic membrane dynamics in malaria parasites
Source: PLoS Pathog. 2026 Sep 28;22(9):e1014608. doi: 10.1371/journal.ppat.1014608 (PMC13645220; doi:10.1371/journal.ppat.1014608)
Supplement: Supplementary file 4 [file ppat.1014608.s012.pdf]

Supplemental Table S4. Transgenic parasite lines used in this study

Mutant	Parental strain[1]	Description
B11	3D7	Constitutive expression of DiCre recombinase
atg18:loxP	B11 (*6*)	Conditional deletion of untagged ATG18 by DiCre
atg18-mNG:loxP	B11 (*6*)	Conditional deletion of mNG-tagged ATG18 by DiCre
atg18:loxP + crt-mNG	*atg18:loxP*	Endogenous mNG-tagging of CRT in ATG18 DiCre line
atg18:loxP + v_1*B-mNG*	*atg18:loxP*	Endogenous mNG-tagging of V_1B in ATG18 DiCre line
atg18:loxP + v_1*C-mNG*	*atg18:loxP*	Endogenous mNG-tagging of V_1C in ATG18 DiCre line
atg18:loxP + v_o*a-mNG*	*atg18:loxP*	Endogenous mNG-tagging of V_oa in ATG18 DiCre line
atg18:loxP + crt-mNG + p40px-mSc	*atg18:loxP + crt-mNG*	Episomal expression of mSc-tagged P40PX in ATG18 DiCre line with mNG-tagged CRT
atg18:loxP + crt-mNG + mSc-rab7	*atg18:loxP + crt-mNG*	Episomal expression of mSc-tagged Rab7 in ATG18 DiCre line with mNG-tagged CRT
atg18:loxP + v_1*B-mNG + crt-mCh*	*atg18:loxP +* v_1*B-mNG*	Endogenous mCh-tagging of CRT in ATG18 DiCre line with mNG-tagged V_1B
atg18:loxP + v_o*a-mNG + crt-mCh*	*atg18:loxP +* v_o*a-mNG*	Endogenous mCh-tagging of CRT in ATG18 DiCre line with mNG-tagged V_oa
atg18-mNG:loxP + crt-mCh	*atg18-mNG:loxP*	Endogenous mCh-tagging of CRT in ATG18-mNG DiCre line
atg18-mNG:loxP + vps26-mCh	*atg18-mNG:loxP*	Endogenous mCh-tagging of Vps26 in ATG18-mNG DiCre line
atg18-mNG:loxP + vps29-mCh	*atg18-mNG:loxP*	Endogenous mCh-tagging of Vps29 in ATG18-mNG DiCre line
atg18-mNG:loxP + ft2-mCh	*atg18-mNG:loxP*	Endogenous mCh-tagging of FT2 in ATG18-mNG DiCre line
atg18-mNG:loxP + pm2-mCh	*atg18-mNG:loxP*	Endogenous mCh-tagging of PM2 in ATG18-mNG DiCre line
atg18-mNG:loxP + hrp2-mCh	*atg18-mNG:loxP*	Endogenous mCh-tagging of HRP2 in ATG18-mNG DiCre line
atg18-mNG:loxP + mSc-rab5a	*atg18-mNG:loxP*	Episomal expression of mSc-tagged Rab5a in ATG18-mNG DiCre line
atg18-mNG:loxP + mSc-rab7	*atg18-mNG:loxP*	Episomal expression of mSc-tagged Rab7 in ATG18-mNG DiCre line
V_o*c:loxP + crt-mNG +* $gbp130_{1\text{-}180}$*-mSc*	V_o*c:loxP + crt-mNG (5)*	Episomal expression of mSc-tagged $GBP130_{1\text{-}180}$ in V_oc DiCre line with CRT-mNG

[1] Recipient strains used as genetic background for transfections.
