# Supplementary material for: PfATG18 links V-ATPase assembly to endocytic membrane dynamics in malaria parasites
Source: PLoS Pathog. 2026 Sep 28;22(9):e1014608. doi: 10.1371/journal.ppat.1014608 (PMC13645220; doi:10.1371/journal.ppat.1014608)
Supplement: Supplementary file 5 [file ppat.1014608.s014.pdf]

SUPPLEMENTAL REFERENCES

1. C. S. Hughes *et al.*, Ultrasensitive proteome analysis using paramagnetic bead technology. *Mol Syst Biol* **10**, 757 (2014).
2. U. Distler, M. Sielaff, S. Tenzer, Label-free proteomics of quantity-limited samples using ion mobility-assisted data-independent acquisition mass spectrometry. *Methods Mol Biol* **2228**, 327-339 (2021).
3. F. Meier *et al.*, diaPASEF: parallel accumulation-serial fragmentation combined with data-independent acquisition. *Nat Methods* **17**, 1229-1236 (2020).
4. V. Demichev, C. B. Messner, S. I. Vernardis, K. S. Lilley, M. Ralser, DIA-NN: neural networks and interference correction enable deep proteome coverage in high throughput. *Nat Methods* **17**, 41-44 (2020).
5. A. Alder *et al.*, The role of *Plasmodium* V-ATPase in vacuolar physiology and antimalarial drug uptake. *Proc Natl Acad Sci U S A* **120**, e2306420120 (2023).
6. A. J. Perrin *et al.*, The actinomyosin motor drives malaria parasite red blood cell invasion but not egress. *mBio* **9**, e00905-00918 (2018).
