# Supplementary material for: PfATG18 links V-ATPase assembly to endocytic membrane dynamics in malaria parasites
Source: PLoS Pathog. 2026 Sep 28;22(9):e1014608. doi: 10.1371/journal.ppat.1014608 (PMC13645220; doi:10.1371/journal.ppat.1014608)
Supplement: Supplementary file 6 [file ppat.1014608.s016.pdf]

SUPPLEMENTAL METHODS

Proteolytic digestion of immunoprecipitated proteins

Eluted proteins were digested on a Biomek i7 robotic pipetting platform (Beckman Coulter Life Sciences) using an adapted single-pot solid-phase-enhanced sample preparation protocol (*1*). Digestion steps followed the protocol from Distler et al., 2021 (*2*) with minor modifications. Proteins were reduced with 20 mM dithiothreitol (DTT, Carl Roth, 6908.2) for 30 minutes at 45°C, then alkylated with iodoacetamide (IAA, Merck, I1149) for 30 minutes at room temperature, and excess IAA was quenched with DTT. Afterwards, 2.5 µg of carboxylate-modified paramagnetic beads (Cytiva, 45152105050250, 65152105050250) were added, as described previously (*1*). Samples were adjusted to a final concentration of 70% acetonitrile (ACN, Carl Roth, NH40.1) and incubated with gentle shaking for 20 minutes. Beads were immobilized by incubation on a magnetic rack for 2 minutes, washed four times with 70% ethanol (Carl Roth, 1HP8.1) and once with ACN, and resuspended in 50 mM NH_4HCO_3 (Carl Roth, T871.1) containing 0.4 µg trypsin (Promega, V5280). Digestion proceeded overnight at 37°C and was stopped by acidification with trifluoroacetic acid (TFA, Fisher Scientific, A116-50).

Peptides were desalted using 25 mg Sep-Pak tC18 µElution plates (Waters, 186002319). Solid-phase extraction resin was conditioned with 200 µL methanol (Merck, 1.060.352.500), followed by rinses with 200 µL of 80% ACN and 0.1% TFA. Peptides were then loaded onto the C18 columns followed by washing with 1 mL of 0.1% TFA. Peptides were eluted with 50 µL of 80% ACN and 0.1% TFA. Desalted peptides were frozen at -80°C, lyophilized, and subsequently resuspended in 20 µL 0.1% formic acid (FA, Fisher Scientific, A117-50) for LC-MS analysis.

Liquid chromatography – mass spectrometry

Samples were analyzed using a nanoElute liquid chromatography system coupled online to a timsTOF Pro mass spectrometer (both from Bruker). Peptides were separated at 400 nL per minute on a heated (50°C) reversed-phase Aurora Ultimate C18 UHPLC emitter column (25 cm x 75 µm, 1.7 µm, IonOpticks, AUR4-25075C18-CSI) in direct injection mode at 600 bar. Mobile phase A was 0.1% FA in water (Merck, 1037281002), and mobile phase B 0.1% FA in ACN. Separation was achieved with a linear gradient from 2 to 37% B over 39 minutes, followed by a 5-minute rinse at 95% B. Eluting peptides were analyzed in positive mode ESI-MS using parallel accumulation serial fragmentation enhanced data-independent acquisition (diaPASEF) (*3*). The dual TIMS operated at near 100% duty cycle with equal accumulation and ramp times of 100 ms each across a mobility range of $1/K_0$ = 0.6-1.6 Vs cm^{-2}. A total of 36 x 25 Th isolation windows from *m/z* 300-1,165 were defined, yielding 2-3 diaPASEF scans per TIMS cycle and an overall cycle time of 1.7 seconds. Collision energy was ramped linearly with mobility from 59 eV at $1/K_0$ = 1.3 Vs cm^{-2} to 20 eV at $1/K_0$ = 0.85 Vs cm^{-2}. Samples were analyzed in triplicate.

Processing of raw mass spectrometry data

Raw data were processed using DIA-NN (version 2.2.0) (*4*) with default settings for library-free database search. A custom database was compiled including the UniProtKB *P. falciparum* reference proteome (March 2025 release, 5,495 entries) and common contaminants. Trypsin was specified as the protease, allowing one missed cleavage. Carbamidomethylation was set as a fixed modification, with no variable modifications allowed. Peptide lengths were restricted to 7-30 amino acids. The precursor and product ion *m/z* ranges were 300-1,800 and 200-1,800, respectively. Quantification was performed in "QuantUMS (high precision)" mode without normalization. MS1 and MS2 mass accuracies, as well as scan window sizes, were optimized using DIA-NN's built-in algorithms, maintaining peptide precursor false discovery rates below 1%.

Statistical downstream analysis of proteomic data

Proteins had to be identified by at least two peptides to be included in the final dataset (see Source Data). Statistical analysis was performed using Student's t-test, with multiple hypothesis testing corrected by the Benjamini-Hochberg method at a false discovery rate of 1%. Identified proteins were ranked based on a combined score reflecting both enrichment and statistical significance, calculated as $\log_2$ fold-change multiplied by the negative $\log_{10}$ of the p-value. To account for the higher protein

content in the *Pf*ATG18 group relative to wild-type controls, proteins detected exclusively in the *Pf*ATG18 pull-downs were excluded from ranking.
