# Supplementary material for: PfATG18 links V-ATPase assembly to endocytic membrane dynamics in malaria parasites
Source: PLoS Pathog. 2026 Sep 28;22(9):e1014608. doi: 10.1371/journal.ppat.1014608 (PMC13645220; doi:10.1371/journal.ppat.1014608)
Supplement: Supplementary file 7 [file ppat.1014608.s017.pdf]

UNCROPPED GELS AND BLOTS

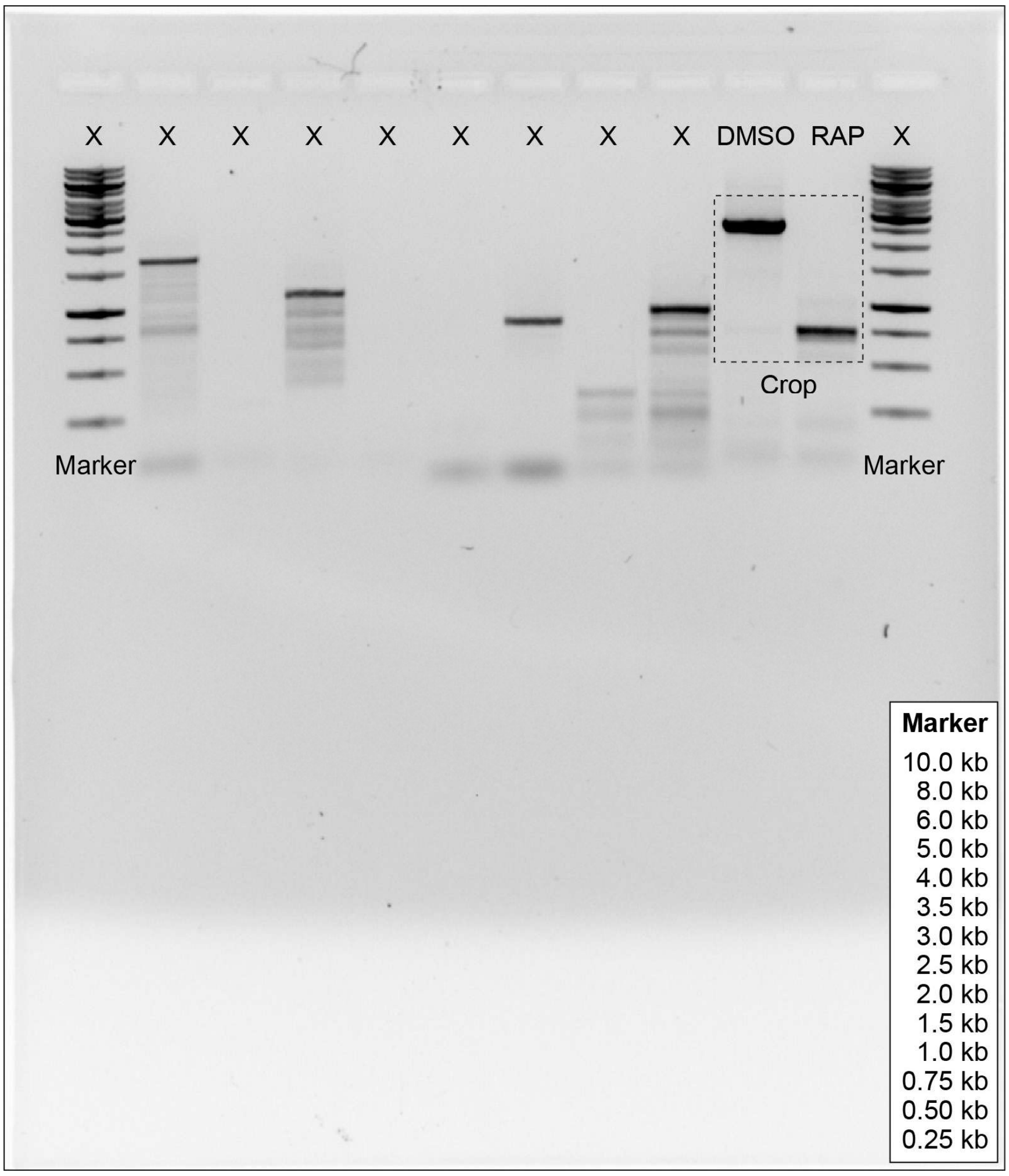

Uncropped DNA gel corresponding to **Figure 2A**. The region used for the figure panel is outlined with a dashed box. Lanes not included in the published figure are marked with an "X". DNA bands were visualized by ethidium bromide staining.

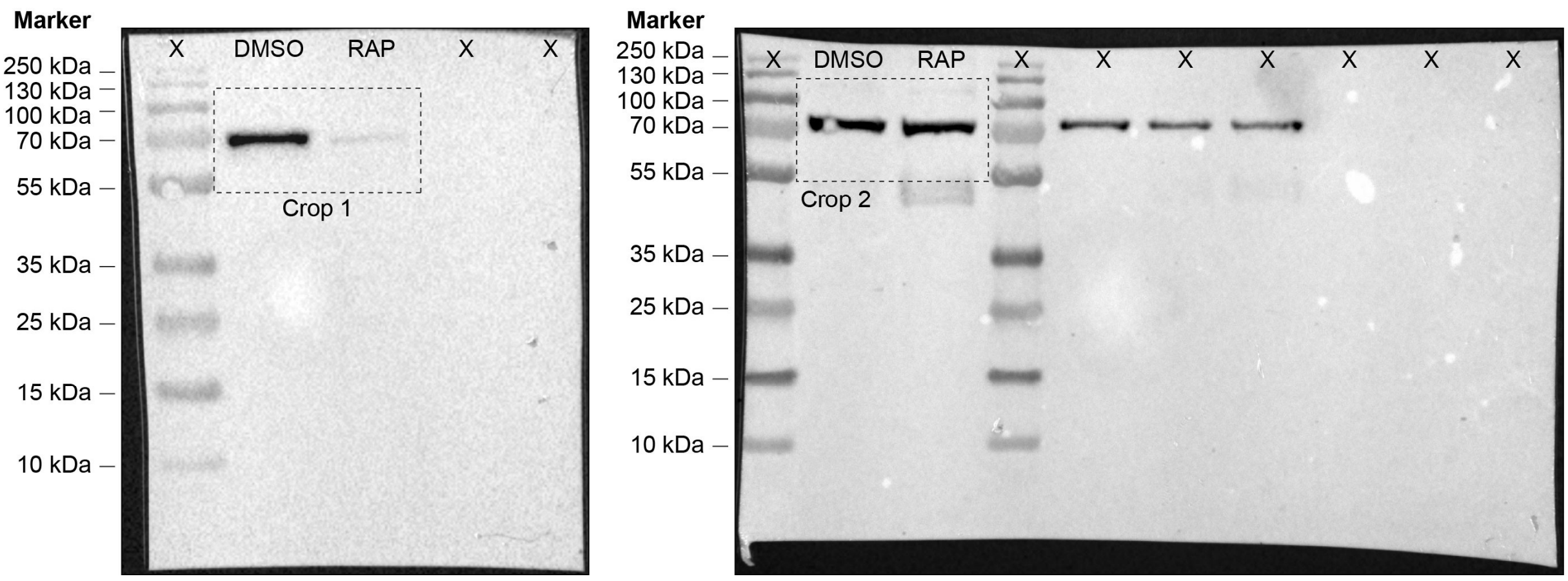

Uncropped Western blots corresponding to **Figure 2B**. The regions used for the figure panel are outlined with dashed boxes. Lanes not included in the published figure are marked with an "X". Protein bands were visualized by chemiluminescence detection.

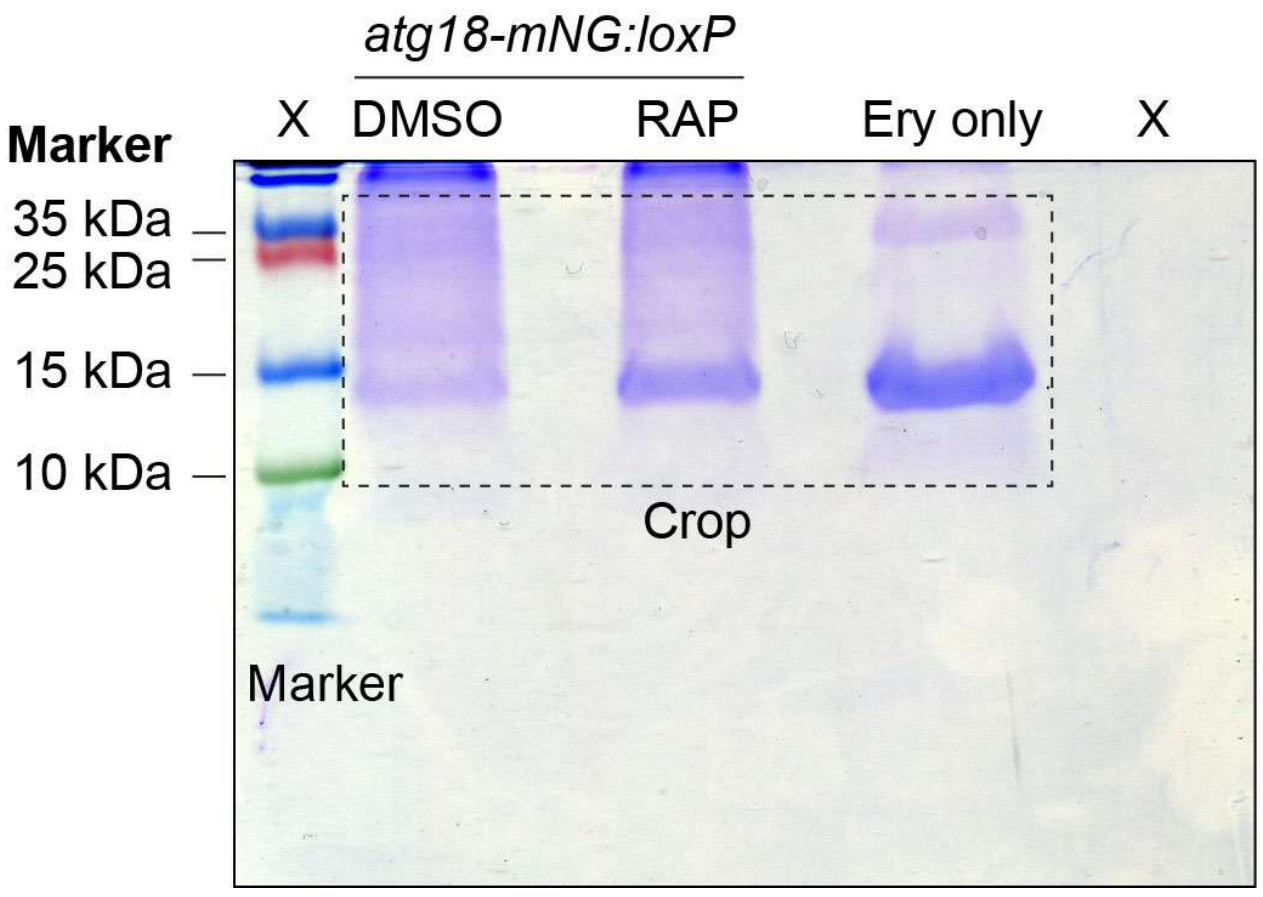

Uncropped polyacrylamide gel corresponding to **Figure 3I**. The region used for the figure panel is outlined with a dashed box. Lanes not included in the published figure are marked with an “X”. Protein bands were visualized by Coomassie Brilliant Blue R-250 staining.

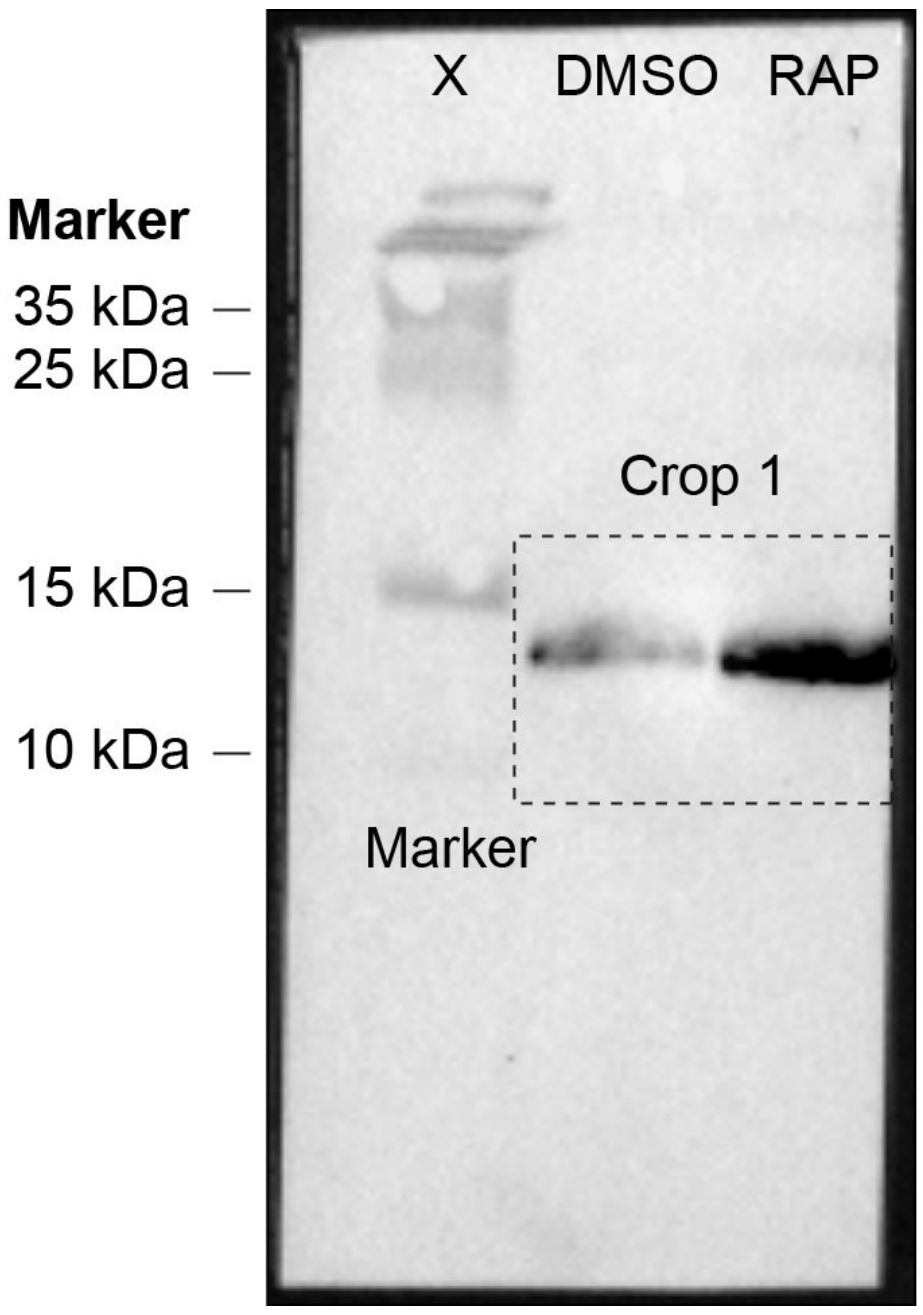

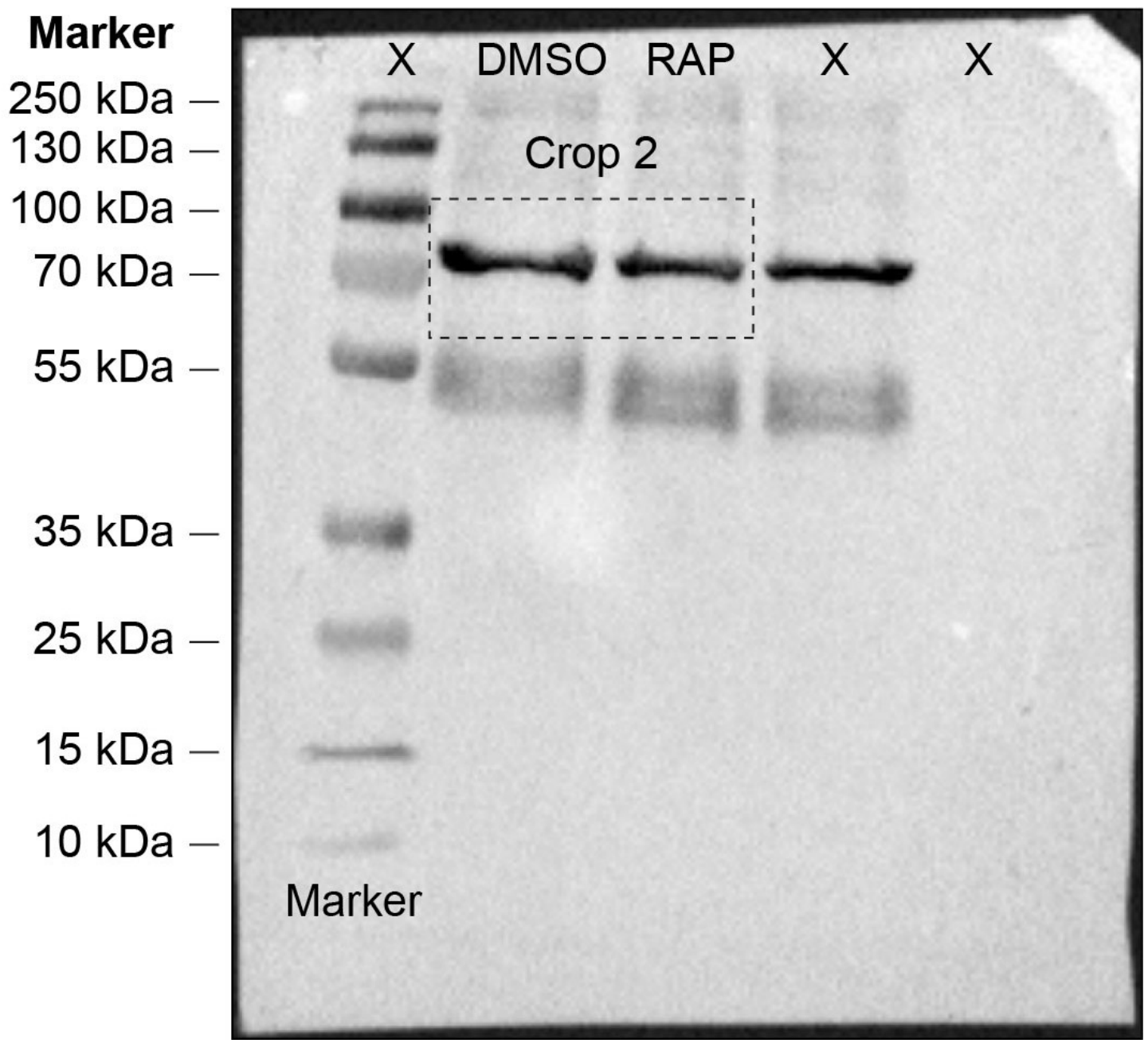

Uncropped Western blots corresponding to **Figure 3J**. The regions used for the figure panel are outlined with dashed boxes. Lanes not included in the published figure are marked with an “X”. Protein bands were visualized by chemiluminescence detection.

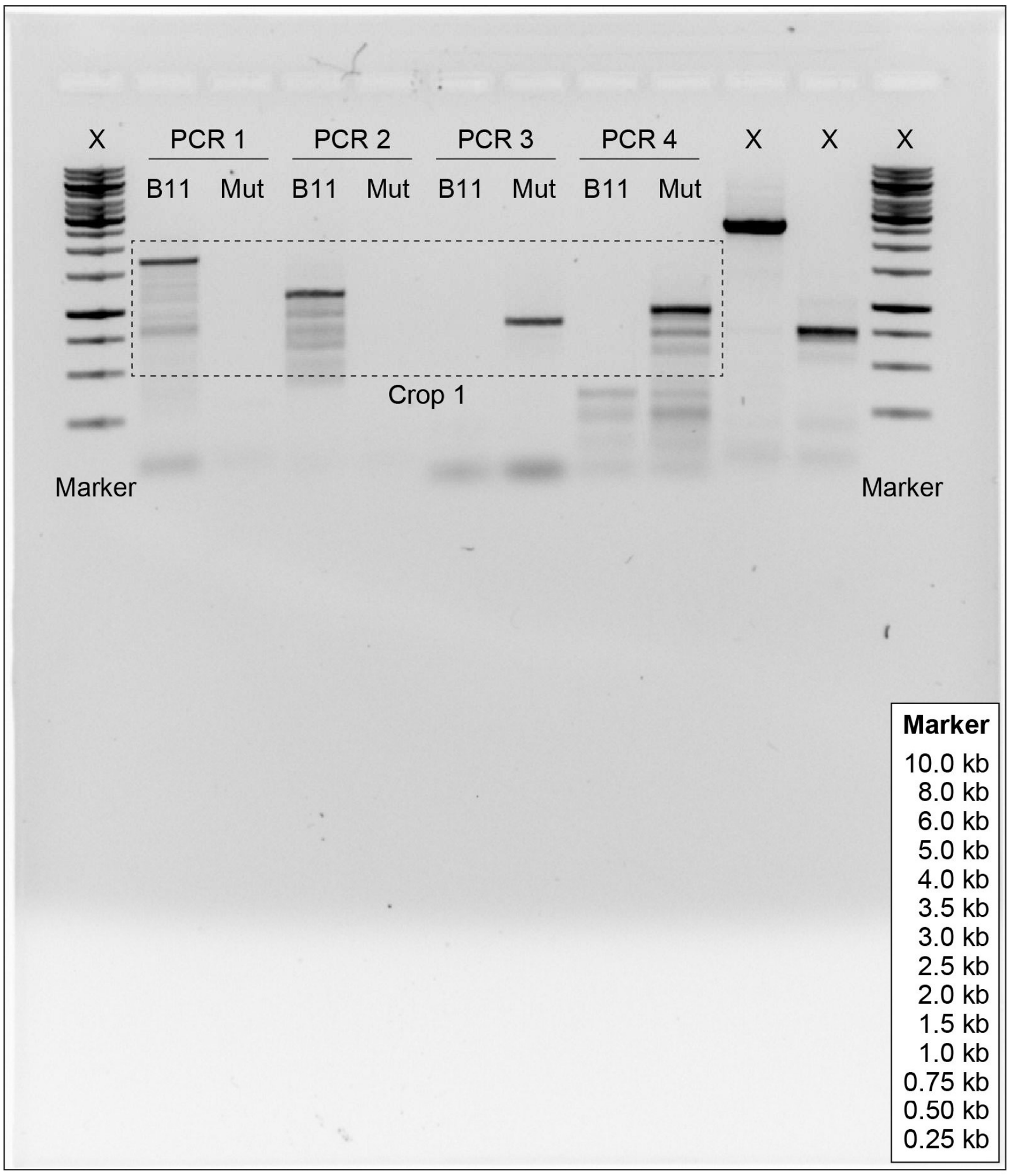

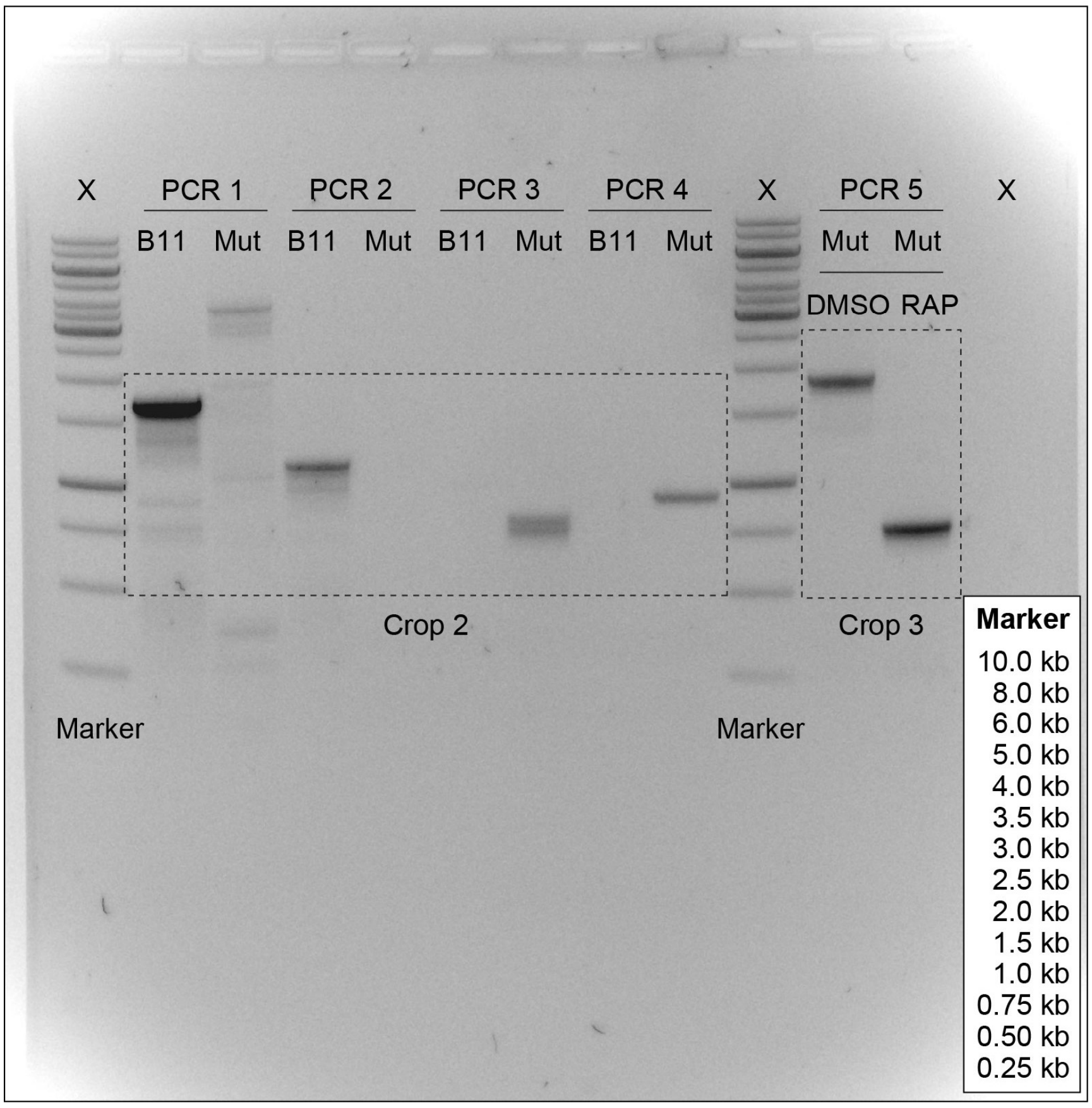

Uncropped DNA gels corresponding to **Figure S1**. The regions used for the figure panel are outlined with dashed boxes. Lanes not included in the published figure are marked with an "X". DNA bands were visualized by ethidium bromide staining.
